# Rational Design of a Glycoconjugate Vaccine against Group A *Streptococcus*

**DOI:** 10.3390/ijms21228558

**Published:** 2020-11-13

**Authors:** Roberta Di Benedetto, Francesca Mancini, Martina Carducci, Gianmarco Gasperini, Danilo Gomes Moriel, Allan Saul, Francesca Necchi, Rino Rappuoli, Francesca Micoli

**Affiliations:** 1 GSK Vaccines Institute for Global Health (GVGH), Via Fiorentina 1, 53100 Siena, Italy; roberta.x.di-benedetto@gsk.com (R.D.B.); francesca.x.mancini@gsk.com (F.M.); martina.x.carducci@gsk.com (M.C.); gianmarco.x.gasperini@gsk.com (G.G.); danilo.x.moriel-gomes@gsk.com (D.G.M.); allan.saul@honorary.burnet.edu.au (A.S.); francesca.x.necchi@gsk.com (F.N.); 2 GSK, Via Fiorentina 1, 53100 Siena, Italy; rino.r.rappuoli@gsk.com

**Keywords:** Group A *Streptococcus*, Group A Carbohydrate, glycoconjugate, vaccine, SLO, SpyCEP, SpyAD, carrier protein

## Abstract

No commercial vaccine is yet available against Group A *Streptococcus* (GAS), major cause of pharyngitis and impetigo, with a high frequency of serious sequelae in low- and middle-income countries. Group A Carbohydrate (GAC), conjugated to an appropriate carrier protein, has been proposed as an attractive vaccine candidate. Here, we explored the possibility to use GAS Streptolysin O (SLO), SpyCEP and SpyAD protein antigens with dual role of antigen and carrier, to enhance the efficacy of the final vaccine and reduce its complexity. All protein antigens resulted good carrier for GAC, inducing similar anti-GAC IgG response to the more traditional CRM_197_ conjugate in mice. However, conjugation to the polysaccharide had a negative impact on the anti-protein responses, especially in terms of functionality as evaluated by an IL-8 cleavage assay for SpyCEP and a hemolysis assay for SLO. After selecting CRM_197_ as carrier, optimal conditions for its conjugation to GAC were identified through a Design of Experiment approach, improving process robustness and yield This work supports the development of a vaccine against GAS and shows how novel statistical tools and recent advancements in the field of conjugation can lead to improved design of glycoconjugate vaccines.

## 1. Introduction

Group A *Streptococcus* (GAS) causes a diverse spectrum of diseases, from superficial infections (pharyngitis, skin infections, cellulitis) to severe invasive diseases (puerperal sepsis, necrotizing fasciitis, streptococcal toxic shock syndrome), with a high frequency of serious sequelae in low- and middle-income Countries (LMICs) (acute rheumatic fever, ARF; rheumatic heart disease, RHD and glomerulonephritis) [1].

Pharyngitis is the most frequent symptomatic GAS infection in children across the world, with more than 400 million cases estimated annually [2] and an important driver of antibiotic use [3] that can ultimately result in increased antimicrobial resistance, a growing public health crisis [4]. Pharyngitis could lead to RHD, which is a chronic inflammatory heart valve condition representing the main global burden of GAS. In 2015, 319 thousand deaths due to RHD were estimated, with >33 million RHD cases and 10 million disability-adjusted life-years (DALYs) lost [5]. Vaccination is the most practical strategy to reduce global GAS associated disease burden in the long term. However, no commercial vaccine is still available against this pathogen [6].

Group A Carbohydrate (GAC) is a surface polysaccharide comprising of a polyrhamnose backbone with alternating *N*-acetylglucosamine (GlcNAc) at the side chain. It represents an attractive vaccine candidate as it is highly conserved and expressed across GAS strains. Indeed, one of the major obstacles for vaccine strategy development is represented by GAS serotype diversity related to other non-carbohydrate antigens [7]. 

Conjugation of polysaccharides (PS) to appropriate carrier proteins is a common procedure for improving their immunogenicity [8]. PS are typical T-cell independent antigens naturally containing only B-cell epitopes and lacking T-cell epitopes. Covalent conjugation to a protein as a source of T-cell epitopes, converts the PS into a T-dependent antigen, with enhanced memory response, class-switching and antibody production in infants [9,10].

It has been reported that human anti-GAC sera successfully promoted phagocytosis of several GAS strains [11], while mice immunized with GAC conjugated to tetanus toxoid (TT) or CRM_197_ carrier proteins were protected against GAS challenges [12,13]. Moreover, an inverse relationship between high anti-GAC antibody titers and the presence of GAS in the throat of Mexican children was evidenced [13].

Chemical conjugation of a PS to a carrier protein is a complex process that can result in the lack of reproducibility and consistency if not performed under robust conditions. Here, a Design of Experiment (DoE) approach was used to identify optimal reaction conditions for linkage of GAC to CRM_197_, improving process robustness and yield.

Conserved protein antigens are also in vaccine development against GAS. In particular, Streptolysin O (SLO), SpyAD and SpyCEP were identified as promising vaccine candidates through a reverse vaccinology approach [14]. SLO has been shown to be a key virulence factor of GAS by preventing internalization of the bacteria into lysosomes where they can be destroyed [15]. Moreover, SLO promotes GAS resistance to phagocytic clearance by neutrophils, facilitating GAS escape from innate immune killing and an inactivated SLO demonstrated to be protective in a murine model against GAS challenge [16]. SpyAD is a surface-exposed adhesin that mediates GAS interaction with host cells. Moreover, deletion of SpyAD gene in a GAS strain led to an impaired capacity of the knockout mutant to properly divide, suggesting also an important role in bacterial division [17]. Finally, SpyCEP is a multi-domain proteinase, with a catalytic domain responsible for the interleukin (IL)-8 and other chemokines cleavage. Cleavage of IL-8 represents a mechanism of immune evasion, preventing IL-8 C-terminus-mediated endothelial translocation and subsequent recruitment of neutrophils [18,19].

These three protein antigens are highly conserved and prevalent in clinical collections and, together with GAC, could virtually cover all GAS clinical isolates [20]. Thus, the formulation of a multicomponent vaccine composed of recombinant SLO, SpyAD and SpyCEP with GAC-CRM_197_ conjugate has been proposed [6]. 

Here, the possibility to use one of the GAS proteins with dual role of antigen and carrier for GAC was tested, aiming to reduce the complexity of the final vaccine formulation. Moreover, CRM_197_ is one of the few carrier proteins currently used in licensed glycoconjugate vaccines against bacterial infections. For this reason, there is increased concern that pre-exposure or co-exposure to this carrier could lead to immune interference and reduction of the anti-carbohydrate immune response [9], thus driving to the need of identifying alternative carrier proteins [21,22].

This work supports the design of an improved vaccine against GAS and represents an example of more rational design of a glycoconjugate vaccine based on the use of novel statistical tools and recent advancements in the field of conjugation.

## 2. Results

### 2.1. Testing Random and Selective Conjugation Chemistries for Linkage of GAC to CRM_197_

Two different approaches were compared for conjugation of GAC to CRM_197_, one of the most extensively and successfully carrier proteins used in glycoconjugate vaccines [21]. The selective direct reductive amination between the aldehyde group at the reducing residue of GAC and lysines of the carrier protein [12] resulted in a conjugate characterized by GAC to CRM_197_ *w*/*w* ratio of 0.18, corresponding to an average of 1.5 chains of GAC per molecule of the carrier. When we produced additional conjugate lots by using the same conjugation conditions, there was large batch-to-batch inconsistency with GAC to CRM_197_ ratios ranging between 0.01 and 0.18. Moreover, in some occasions, no conjugate formation was verified.

**Figure 1:**
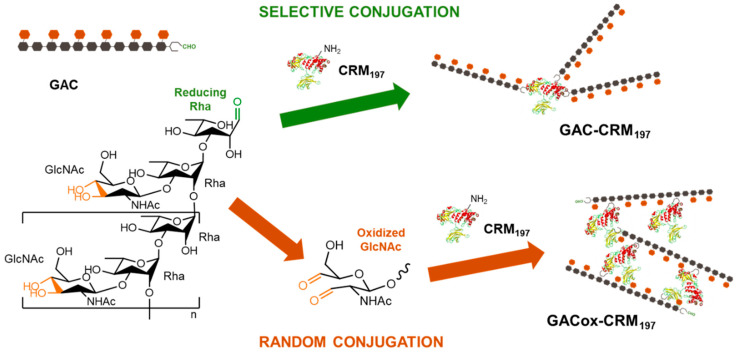
Conjugation strategies for producing Group A *Streptococcus* (GAS) conjugates: selective direct reductive amination between the aldehyde group at the reducing residue of GAC and lysines of the carrier protein (green approach) [12] and reductive amination between the aldehyde groups randomly generated through oxidization of GAC and lysines of the carrier protein (orange approach).

An alternative random approach was tested, that still relies on a reductive amination chemistry. In particular, a step of random GAC oxidation with sodium periodate was introduced, producing additional aldehydic groups along the polysaccharide chains. The oxidation occurs at the vicinal diols of the GlcNAc side chain of GAC. The reductive amination of oxidized GAC was performed with the same conditions used for linkage of GAC via its reducing end (GAC concentration of 10 mg/mL, GAC to CRM_197_ to NaBH_3_CN *w*/*w*/*w* ratio of 4:1:2, 200 mM phosphate buffer at pH 8, 2 days at 37 °C), resulting in a conjugate with GAC to CRM_197_ *w*/*w* ratio of 0.2, similar to that of the selective conjugate. The two conjugation schemes are reported in Figure 1.

**Figure 2:**
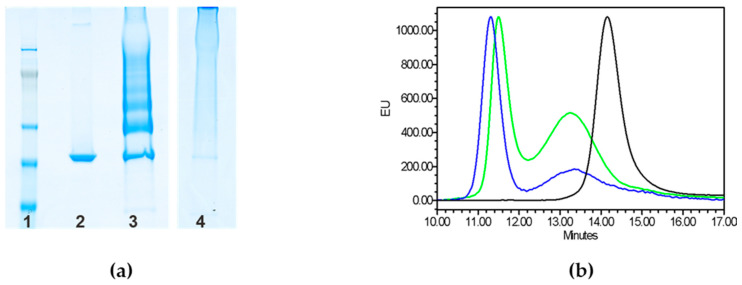
(**a**) Characterization by sodium dodecyl sulfate-polyacrylamide gel electrophoresis (SDS-PAGE) analysis (7% Tris-acetate gel) of the conjugation mixtures in comparison to unconjugated CRM_197_. Ten µg of conjugated protein and 2 µg of unconjugated CRM_197_ were loaded per well. Lane 1: marker, lane 2: CRM_197_, lane 3: selective GAC-CRM_197_, lane 4: random GACox-CRM_197_. (**b**) HPLC-SEC profiles (fluorescence emission detection) of selective GAC-CRM_197_ conjugation mixture (blue line), random GACox-CRM_197_ conjugation mixture (green line) and unconjugated CRM_197_ (black line), 80 µL of sample injected on a TSK gel G3000 PW_XL_ column; 0.1 M NaCl 0.1 M NaH_2_PO_4_ 5% CH_3_CN pH 7.2 at 0.5 mL/min. Vtot 23.326 min, V0 10.663 min.

As expected, random and selective conjugates showed a different protein pattern by sodium dodecyl sulfate-polyacrylamide gel electrophoresis (SDS-PAGE) analysis: single bands at increasing molecular weight (MW) for the selective approach, corresponding to increasing number of GAC chains linked to CRM_197_ vs. a polydisperse smear at very high MW for the random conjugate (Figure 2a). Conjugate formation was also confirmed by High Performance Liquid Chromatography–Size Exclusion Chromatography (HPLC-SEC) (Figure 2b). The profiles of the two conjugates differed significantly from SDS-PAGE patterns. In fact, differently from what expected, the random conjugate showed a main peak at slightly higher retention time compared to the selective conjugate. Indeed, HPLC-SEC estimates an apparent MW that can reflect the different structure of the two constructs. HPLC-SEC analysis also confirmed absence of free CRM_197_ in both conjugation mixtures. Residual unconjugated GAC was removed by size exclusion chromatography on Sephacryl S-100 HR column. Total GAC recoveries after purification were approximately 5% for both conjugates.

**Figure 3:**
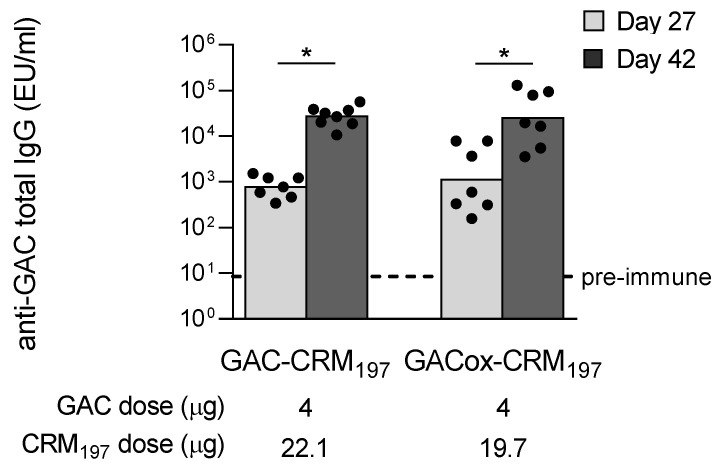
Immunogenicity of GAC when conjugated to CRM_197_ through different chemistries. CD1 mice were immunized intraperitoneally (i.p.) at day 0 and 28 with 4 µg GAC/dose formulated with 2 mg/mL Alhydrogel. Summary graph of anti-GAC specific IgG geometric mean units (bars) and individual antibody levels (dots) is reported (GAC-HSA used as coating antigen). Not enough sera was available at day 27 for one mice immunized with GAC-CRM_197._ Mann-Whitney two-tailed test was performed to compare the response induced by the two immunization groups (*p* > 0.05) whereas Wilcoxon test was performed to compare the responses for each group at day 27 and day 42 (* *p* < 0.05).

The two conjugates produced via random and selective approaches were compared in mice, to check if random linkage of GAC to the protein could negatively impact on the induced immune response. Both conjugates induced no significant different anti-GAC IgG response 4 weeks after the first immunization, with similar booster (*p* < 0.05) 2 weeks after the second dose (Figure 3). Similar anti-CRM_197_ IgG responses were also induced (Supplementary Figure S1).

### 2.2. Applying Random Chemistry for Linkage of GAC to GAS Proteins

To increase GAC recovery, the reductive amination step conditions were slightly modified (GAC concentration increased from 10 to 40 mg/mL, GAC to CRM_197_ to NaBH_3_CN *w*/*w*/*w* ratio of 4:1:2, borate buffer at pH 8 instead of phosphate [23], 2 days at 37 °C), resulting in a conjugate with GAC/CRM_197_ *w*/*w* ratio increased from 0.2 to 0.86 and GAC yield from 5% to 21.5%.

The same conditions were applied for linking GAC to GAS SLO, SpyAD and SpyCEP protein antigens. However, because the melting temperature (Tm) by Differential Scanning Calorimetry (DSC) for these proteins resulted to be close to 37 °C (Tm of 39.35 °C for SLO, 44.37 °C for SpyAD, 40.03 °C for SpyCEP), the reactions were performed at 25 °C instead of 37 °C, trying to preserve GAS proteins folding and, possibly, functionality in the final conjugates.

**Figure 4:**
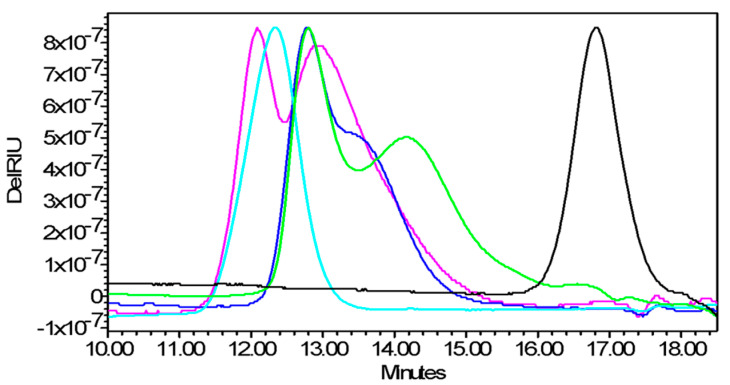
High Performance Liquid Chromatography–Size Exclusion Chromatography (HPLC-SEC) profiles (refractive index detection) of GACox-CRM_197_ (green line), GACox-SLO (light blue line), GACox-SpyAD (blue line), GACox-SpyCEP (pink line) conjugates compared to unconjugated GAC (black line). 80 µL of sample injected on a TSK gel G3000 PW_XL_ column; 0.1 M NaCl 0.1 M NaH_2_PO_4_ 5% CH_3_CN pH 7.2 at 0.5 mL/min. Vtot 23.326 min, V0 10.663 min.

Conjugate formation was confirmed by SDS-PAGE for all the conjugates, also revealing absence of free proteins (Supplementary Figure S2). Purification by Amicon 30 kDa cut-off successfully reduced level of free GAC to <10% for all conjugates, as verified by HPLC-SEC (refractive index detection) (Figure 4), also confirming conjugate formation. The conjugates were characterized by a similar GAC/protein molar ratio, higher than with SLO (Table 1).

**Figure 5:**
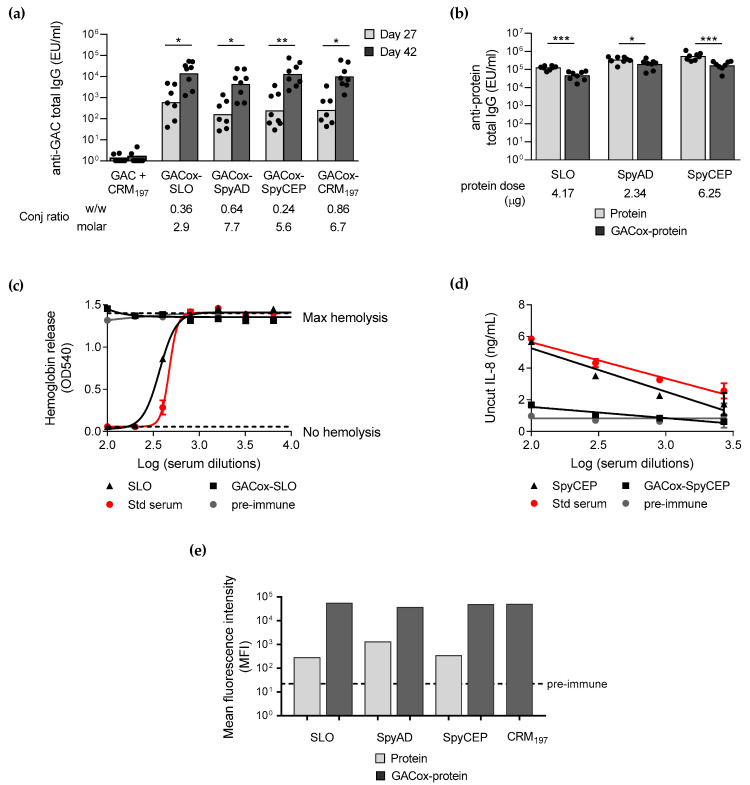
Immunogenicity of GAC physically mixed to CRM_197_ and when conjugated to CRM_197_ or GAS proteins SLO, SpyAD and SpyCEP. CD1 mice were immunized intraperitoneally (i.p.) at day 0 and 28 with 1.5 µg GAC/dose or with the corresponding dose of the carrier protein alone, all formulated with 2 mg/mL Alhydrogel. Sera were analyzed by enzyme-linked immunosorbent assay (ELISA) using as coating antigens GAC-HSA (**a**) or SLO, SpyAD and SpyCEP (**b**). Summary graphs of anti-antigen specific IgG geometric mean units (bars) and individual antibody levels (dots) are reported. Not enough sera was available for one mice of day 27 for GACox-SLO, GACox-SpyAD and GACox-CRM_197_ groups. Kruskal-Wallis test was performed among the 4 groups immunized with the conjugates in graph (**a**) (*p* > 0.05), Wilcoxon test was performed between response at day 27 and day 42 in graph (**a**) and Mann-Whitney two-tailed test between each group immunized with protein alone or GACox-protein conjugate in graph (**b**) (* *p* < 0.05, ** *p* < 0.01, *** *p* < 0.001). Sera were tested in the hemolysis inhibition assay (**c**) and in the IL-8 cleavage inhibition assay (**d**) to evaluate their ability to block native SLO and SpyCEP activity, respectively. The amount of hemoglobin released by rabbit red blood cells (**c**) and of uncut IL-8 (**d**) observed at each serum dilution tested is reported for pre-immune serum, standard serum and one selected day 42 serum for each immunization group. Each serum dilution was tested twice and the mean value with error bar was reported in the graph (for some points the error bars are not shown as shorter than the height of the symbol). Pooled sera at day 42 were tested in Flow cytometry (FACS) (**e**) to evaluate their ability to bind to GAS bacterial cells. Following incubation of bacteria with the different sera, APC-conjugated anti-mouse IgG secondary antibody was used for detection. The mean fluorescence intensity (MFI) of 10,000 acquired events, measured for each serum, is reported as compared to pre-immune sera. Results obtained with sera diluted 1:500 are reported, as representatives of the three dilutions tested (1:500, 1:5000 and 1:10,000).

When compared in mice, the conjugates with the GAS protein antigens induced same anti-GAC IgG response compared to GAC-CRM_197_ both 4 weeks after first and 2 weeks after second injection, showing that all GAS proteins tested were good carriers for GAC. All conjugates were able to elicit a booster response after re-injection (Figure 5a). Importantly, the physical mixture of GAC with one of the carrier proteins tested did not give a significant anti-GAC IgG response, confirming the role of the carrier protein at inducing T-cell activation and isotype switching.

A Flow cytometry analysis (FACS) against GAS bacterial cells was performed with pooled sera collected 2 weeks after the second injection from each immunization group (Figure 5e). Antibodies induced by all conjugates were able to similarly bind the bacterial cells. Sera induced by unconjugated GAS proteins bound GAS bacteria to a less extent compared to the corresponding conjugates.

However, when GAS proteins were used as carrier, anti-protein-specific total IgG decreased if compared with immunization with the same dose of unconjugated protein (Figure 5b). The effect of conjugation of GAC to GAS proteins was evident when serum functionality was analyzed. Conjugation of GAC completely abolished the ability of SLO and SpyCEP to elicit antibodies able to block native SLO hemolytic activity (Figure 5c) and native SpyCEP protease activity (Figure 5d), respectively.

**Figure 6:**
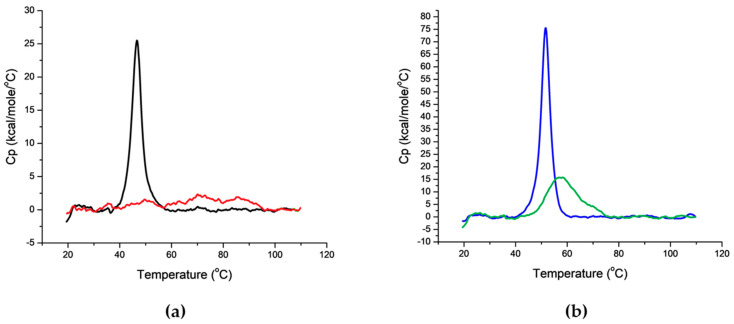
Differential Scanning Calorimetry (DSC) thermograms of (**a**) unconjugated SLO vs. GACox-SLO and (**b**) unconjugated SpyAD vs. GACox-SpyAD. GAS proteins and corresponding conjugates were analyzed in phosphate buffer at pH 7.2, at the same molar concentration of 3 µM for SLO and 2 µM for SpyAD. The ∆H value (from the integrated areas under the curves) for each thermogram was: 1.3 × 10^5^ kcal/mole (SLO, black line); nd (GACox-SLO, red line); 3.7 × 10^5^ kcal/mole (SpyAD, blue line); 2.4 × 10^5^ kcal/mole (GACox-SpyAD, green line).

Through a Differential Scanning Calorimetry (DSC) analysis, strong impact of conjugation on SLO and SpyAD folding was verified, probably correlated with the loss of functionality evidenced. For SLO, folding was not retained at all after conjugation, whereas for SpyAD a decrease in the enthalpy change (ΔH) was observed (Figure 6).

Thus, based on the results obtained, CRM_197_ was selected as the best carrier for GAC and the conjugation process was further optimized through a DoE approach with the main aim to maximize GAC yield and assure robustness of the process.

### 2.3. Optimization of the Random Chemistry through a DoE Approach

#### 2.3.1. Identification of Optimal Conditions for GAC Oxidation

After performing some preliminary experiments, a first DoE was performed to understand which parameters could affect the GAC oxidation step, aiming at identifying their best combination to obtain optimal oxidation degree for efficient conjugation, preventing major impact on GAC structural integrity.

A full factorial, response surface design, with alpha of 1.68179 (rotatable), with 1 replicate of axial and factorial points and 6 center point replicates, was used.

GAC concentration in the range 1–10 mg/mL, pH in the range 5–8 and NaIO_4_ concentration in the range 0.5–10 mM were the factors evaluated. Reaction time and temperature were set, respectively, at 30 min and 25 °C. Conditions used for oxidation and results are summarized in Supplementary Table S1.

Similar GAC recoveries were obtained in all reaction conditions. We also verified no impact on polysaccharide chain length, as expected as GlcNAc, that is the sugar impacted by the oxidation, is in the side chain and not in the backbone of GAC.

In the design space tested, the % GlcNAc oxidation was in the range 8.5–19.4%, meaning that a maximum of 3 repeating units as average per PS chain were oxidized (considering an average of 14 repeating units per GAC chain).

To elaborate the data, a response surface with a quadratic model was chosen and with a backward elimination process, the non-significant terms (*p*-value > 0.05) were removed from the model (statistical analysis in Supplementary Figure S3). The residuals (externally studentized) were normally distributed (Anderson-Darling normality test, *p* = 0.837) and the model resulted with an adjusted-R2 of 0.71.

**Figure 7:**
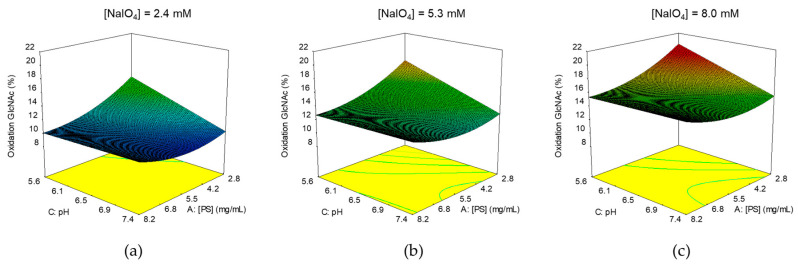
Identification of optimal conditions for GAC oxidation: 3D Surface Model Graphs for % GlcNAc oxidation response. Correlation between pH and GAC concentration at NaIO_4_ concentration of 2.4 (**a**), 5.3 (**b**), 8.0 (**c**).

The GlcNAc oxidation response was affected by all factors investigated and mainly by NaIO_4_ concentration (*p* = 0.0003) (Figure 7).

From the model we achieved a target of oxidation of 15%. Working at pH 8, allowed quenching of NaIO_4_ excess with Na_2_SO_3_ and the subsequent conjugation without GACox intermediate purification. By fixing the pH at 8, the target oxidation level could be reached by working with 8 mM NaIO_4_, quite independently from GAC concentration in the range investigated.

#### 2.3.2. Identification of Optimal Conditions for GAC Conjugation to CRM_197_

After having identified optimal conditions for GAC oxidation, the DoE approach was used to understand which parameters are critical for the conjugation step and to identify their optimal combination to maximize GAC yield, ensuring robustness of the process. 

A full factorial, response surface design, with alpha of 1.0 (face centered), with 1 replicate of axial and factorial points and 6 center point replicates, was used.

GACox, CRM_197_ and NaBH_3_CN concentrations were the factors evaluated, all tested in the range 10–40 mg/mL. Reaction time, temperature and pH were set, respectively, at 2 days, 25 °C and pH 8 in borate buffer. Conditions used for the conjugation tests and results obtained are summarized in Supplementary Table S2. 

Unconjugated CRM_197_ was >10% only in 3 of the 20 tests performed and absent in 15 of them, as calculated by HPLC-SEC analysis. In the design space tested, GAC/CRM_197_ *w*/*w* ratios were in the range 0.12–0.65, whereas GAC recovery ranging from 9.2 to 41.9%, as calculated by Anion Exchange Chromatography coupled with Pulsed Amperometric Detection (HPAEC-PAD). 

To elaborate the data, for either GAC/CRM_197_ *w*/*w* ratios and GAC yields, a response surface with a linear model was chosen. The non-significant terms (*p*-value > 0.05) were removed from the models using a backward elimination process (statistical analyses in Supplementary Figure S4). The residuals (externally studentized) for both models were normally distributed. Normality was calculated through the Anderson-Darling test (*p* = 0.166 for GAC/CRM_197_ *w*/*w* ratio and *p* = 0.676 for GAC yield) and the models resulted with adjusted-R2 of 0.87 and 0.83 for GAC to protein ratio and GAC recovery, respectively. 

**Figure 8:**
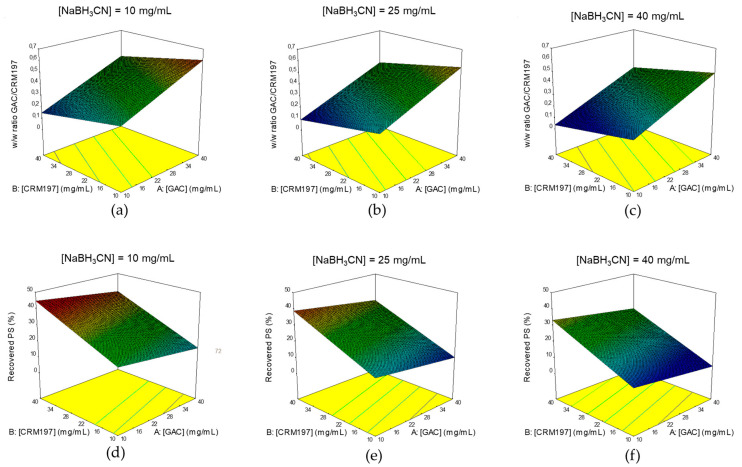
Identification of optimal conditions for GACox conjugation to CRM_197_: 3D Surface Model Graphs for GAC/CRM_197_ *w*/*w* ratio (**a**–**c**) and GAC yield (**d**–**f**) responses. Correlation between CRM_197_ and GAC concentrations at NaBH_3_CN concentrations of 10 (**a**,**d**), 25 (**b**,**e**) and 40 (**c**,**f**).

For both the responses evaluated, all factors investigated in the DoE affected the responses (Figure 8). Interestingly, GAC/CRM_197_ *w*/*w* ratio and GAC recovery increased by reducing NaBH_3_CN concentration.

Based on the results, optimization was done, with all factors in range, maximizing the % of GAC recovery with higher importance than for GAC/CRM_197_ *w*/*w* ratio maximization. 

Optimal conditions identified are reported in Table 2, along with predicted responses and with the actual results obtained by performing the conjugation in the identified reaction conditions. Results obtained were in agreement with those expected, confirming consistency of the process, as all the responses obtained were within the 95% of confidence interval (CI) for Mean.

Having identified NaBH_3_CN concentration as a critical factor for the process, additional conjugation tests were performed further decreasing NaBH_3_CN concentration from 10 to 5 and 1 mg/mL, to check if further lowering the concentration of this reagent could be beneficial to conjugation efficiency. Furthermore, role of reaction time was investigated, running the conjugations at 4 h, overnight (ON) or for 2 days. The other parameters were kept the same, as per DoE optimization. Carrying out the reaction with 5 and 1 mg/mL of reducing agent resulted in conjugates with slightly higher GAC to CRM_197_ ratio compared to 10 mg/mL NaBH_3_CN concentration (Table 3).

**Figure 9:**
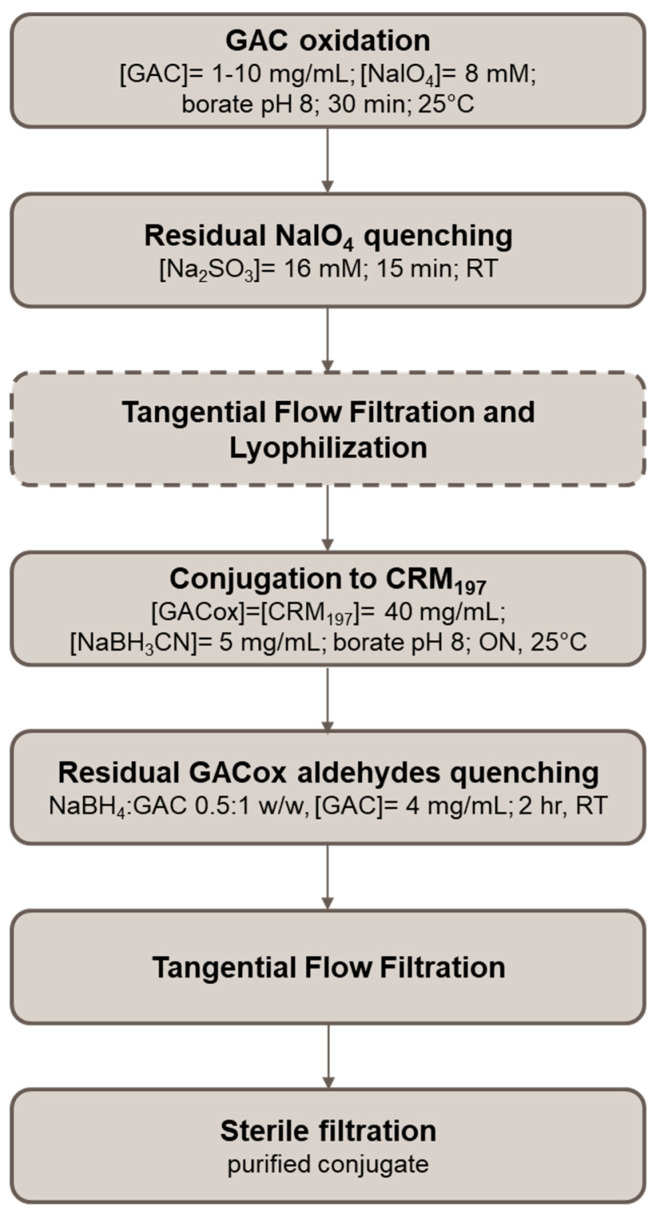
Flow chart of GAC to CRM_197_ optimized conjugation process.

Further reducing NaBH_3_CN concentration from 1 to 0.25 mg/mL negatively impacted on GAC to CRM_197_ ratio and GAC recovery %. Based on such results, 5 mg/mL NaBH_3_CN was selected and reaction time reduced to ON. Optimized conjugation process is described in Figure 9.

The process was also scaled up to 100 mg GAC, further confirming robustness of the process, as the resulting conjugate was characterized by similar GAC to CRM_197_ *w*/*w* ratio and GAC % yield compared to a conjugate produced at 10 mg scale (Table 4) and again the results obtained were within the 95% CI for Mean from the DoE optimization reported in Table 2. 

Such conjugate was tested in mice confirming its ability to induce an anti-GAC IgG response comparable to that elicited by the CRM_197_ conjugate produced before the optimization (Figure 5).

## 3. Discussion

No licensed vaccine is yet available against GAS, a leading cause of global morbidity and mortality worldwide, responsible for a wide range of diseases and estimated to cause about 0.5 million annual deaths, mostly in young adults [2]. One of the main barriers to vaccine development is related to the high GAS strain diversity, serologically based on the serotype of the surface M protein [15], one of the major virulence and immunological determinants of GAS [24]. To date only M protein based candidate vaccines have been tested in clinical trials [6,25,26,27] but novel vaccines based on conserved protein antigens and surface polysaccharide are also in development [28]. The highly conserved SLO, SpyAD, SpyCEP and GAC conjugated to a carrier protein have been proposed as an attractive alternative vaccine candidate [6].

Here, these three protein antigens have been tested as carrier for GAC, with the aim to simplify the final vaccine design, combining two of the four antigens in one construct. To date, only few carrier proteins have been used for licensed glycoconjugate vaccines and there is increased concern for carrier-induced epitope suppression (CIES), that could result in reduced anti-carbohydrate immune response after patient repeated exposure, simultaneously or in close sequence, to a given carrier [21,29,30]. The identification of new carriers is driven also by the interest to explore the dual role as carrier and antigen that a pathogen-related protein can play, thus resulting in a vaccine that, by simultaneous administration of carbohydrate and protein antigens, tackles two different virulence factors of the pathogen [21]. Such type of combinations has already been proposed and investigated at the preclinical level [31,32,33,34,35,36]. Among these, also few GAS proteins have been explored as possible carriers. A variant of GAC chain, conjugated to GAS arginine deiminase (ADI) protein antigen, was able to protect from superficial skin infection but not against invasive GAS disease, in a challenge study in mice [37]. GAC oligosaccharides, conjugated to an inactive mutant of GAS C5a peptidase (ScpA), ScpA193, induced robust anti-carbohydrate immune responses in mice. Antibodies induced mediated GAS opsonophagocytosis in vitro, as well as effectively protected animals from GAS challenges and GAS-induced pulmonary damage. However, anti-ScpA193 antibodies induced by the protein alone had only moderate binding activity to GAS cells and no opsonophagocytic activity, despite the high titers induced [38,39]. 

Either SLO, SpyAD and SpyCEP proteins tested here have proven to be good alternative carriers to the benchmark CRM_197_ for promoting anti-GAC IgG response as well as binding to GAS bacteria by FACS (Figure 5). These results make these proteins attractive as new carrier proteins, potentially to be used also with other PS antigens.

However, anti-protein specific antibodies induced by the conjugates were significantly lower than those induced by the proteins alone, with loss of functionality verified for SLO and SpyCEP (Figure 5). Indeed, in the case of a carrier with a concomitant role of protective antigen, the protein provides not only T-cell helper epitopes but also protective B-cell epitopes. In this case, the extent and location of the saccharide chains on the protein carrier might be relevant in terms of preservation of its key B-cell epitopes [21]. Moreover our results seem to confirm the importance of GAS protein antigens conformation for immunogenicity [40,41], as conjugation impacted on protein folding, as evidenced through DSC analysis, which was probably correlated with the loss of the functionality observed. Alternative conjugation methods, including protein site-selective chemical or enzymatic approaches [42] or bioconjugation [43], could be investigated to preserve protein protective epitopes, without impacting too much on protein structures and conformation. A bioconjugate vaccine produced with *Staphylococcus aureus* type 5 capsular PS (CP5) linked to *S. aureus* α toxin (Hla) has been already shown to be protective against both bacteremia and lethal pneumonia, providing broad-spectrum efficacy against staphylococcal invasive disease, with specific protective antibodies induced against both the glycan and the protein moiety [44]. 

Here, a more traditional semi-synthetic approach was used for conjugation of GAC. It is well known that the conjugation chemistry used, which actually affects the efficiency of conjugation, saccharide to protein ratio and glycoconjugate structure and size, is one of the parameters that can mostly impact the immunogenicity of glycoconjugate vaccines [45,46,47]. We compared terminal linkage of GAC to CRM_197_ with a random approach. Both conjugates elicited similar immune response in mice. In principle, the use of selective chemistry, resulting in more homogeneous and well-defined structures with no impact on sugar chains, should be preferable in terms of production consistency. Nevertheless, in our case the use of the selective approach resulted in batch-to-batch inconsistency with no conjugate formation in some cases. As several details on GAC biosynthetic pathway and anchorage to the peptidoglycan are still speculative [48], it is not clear whether all GAC chains have an available terminal reducing end for conjugation after extraction with nitrite and glacial acetic acid [49,50,51]. This could be an explanation for the conjugation inconsistency observed. Indeed, introduction of few more reactive aldehyde groups along the GAC chain, compared to the aldehyde group on the terminal reducing end of the sugar, allowed more reproducible conjugation. From a process perspective, the synthesis of the random conjugate requires one more step compared to the selective one. By quenching the excess of the oxidizing agent with sodium sulfite, the carrier protein could be directly added in the mixture avoiding GACox intermediate purification and simplifying the process to one step only (Figure 9).

As a random approach leads to the formation of cross-linked and rather undefined and heterogeneous structures, a careful and tight control of the manufacturing process is essential to guarantee consistency together with a proper analytical characterization [46]. 

Here, a DoE approach was used to identify optimal conjugation conditions for assuring process robustness and improving yields. Yield increase means reducing cost-of-goods to have a more sustainable and affordable product, an important matter to meet the vaccination demand in LMICs. 

The DoE methodology, compared to the traditional one-factor-at-a-time (OFAT) approach, allows identification of optimal combination of the critical parameters, considering their interaction and to model the process in the design space investigated, predicting impact of changes in the critical parameters of the quality of the final product [52,53]. In the vaccines field, DoE has been used for development or optimization of analytical and immunological assays [54,55,56] or for improving vaccine formulations or purification processes [57,58,59,60]. 

In our study, DoE has been used for optimizing a conjugation process. Through this approach, conjugation yield has been increased from 5% to around 40% and process robustness has been assured and confirmed, also scaling up the process to 100 mg-scale of GAC. 

In conclusion, this work supports the development of a universal vaccine against GAS and shows how novel tools can be used for the design of improved vaccines, with the final goal to ensure consistent delivery of safe and efficacious products with robust manufacturing processes. Additional studies are ongoing to better evaluate the immune response and protective efficacy induced by the GAC conjugate designed through this work. This was the first step toward a long way that will allow to test finally the conjugate in clinical trials.

## 4. Materials and Methods

### 4.1. Materials

GAC was extracted from a M protein-mutant strain (GAS51∆M1) generated from the wild-type strain HRO-K-51 kindly provided by the University of Rostock. GAS recombinant proteins SpyAD (SpyADstop, 89.5 kDa, 62 lysines in total) and SpyCEP (SpyCEP double mutant, 174.0 kDa, 133 lysines in total) were produced and purified at GVGH as previously described [17,61], GAS recombinant protein SLO (SLO double mutant, 60.6 kDa, 56 lysines in total) and CRM_197_ (58.4 kDa, 39 lysines in total) were obtained from GSK R&D (Siena, Italy).

GAC was chemically extracted from bacterial culture through nitrite/glacial acetic acid treatment [51]. The purification was performed using a combination of tangential flow filtration and anionic exchange chromatography, as previously described [12]. Purified GAC contained no hyaluronic acid, <4% protein and <1% DNA impurities (*w*/*w* with respect to GAC). Average molecular size of 7.0 kDa was estimated by HPLC-SEC analysis (TSK gel G3000 PW_XL_ column) using dextrans (5, 25, 50, 80, 150 kDa) as standards (Merck, Darmstadt, Germany), corresponding to an average of 14 repeating units per chain. 

The following chemicals were used in this study: sodium phosphate monobasic (NaH_2_PO_4_), sodium phosphate dibasic (Na_2_HPO_4_), sodium cyanoborohydride (NaBH_3_CN), sodium periodate (NaIO_4_), sodium sulfite (Na_2_SO_3_), sodium borohydride (NaBH_4_), deoxycholate (DOC), hydrochloric acid (HCl), sodium chloride (NaCl) (Merck, Darmstadt, Germany), boric acid solution, phosphate buffered saline tablets (PBS) (Honeywell Fluka, Charlotte, NC, USA), dithiothreitol (DTT) (Invitrogen, Waltham, MA, USA).

### 4.2. Conjugation of GAC to CRM_197_ through Selective Direct Reductive Amination

The conjugation was performed as reported by Kabanova et al. [12]. Briefly, the reaction was carried out in 200 mM phosphate buffer (NaPi) at pH 8, with GAC concentration of 10 mg/mL and a *w*/*w*/*w* ratio of GAC to CRM_197_ to NaBH_3_CN of 4:1:2. After 2 days at 37 °C the conjugate was purified by size exclusion chromatography on a 1.6 × 60 cm Sephacryl S-100 HR column (Cytiva Life Sciences, Marlborough, MA, USA; formerly GE Healthcare Life Sciences) eluted at 0.5 mL/min in 10 mM NaPi pH 7.2. Final purified conjugate was designated as GAC-CRM_197_.

### 4.3. Conjugation of GAC to Different Carrier Proteins through Random Oxidation Followed by Reductive Amination

#### 4.3.1. GAC Oxidation

After optimization of this step through DoE, GAC 1–10 mg/mL was oxidized with 8 mM NaIO_4_ in borate at pH 8. The solution was kept at 25 °C in the dark, for 30 min. After that, NaIO_4_ excess was quenched with 16 mM Na_2_SO_3_ in borate at pH 8. The mixture was gently stirred at room temperature (RT) for 15 min. The mixture was directly used for conjugation without intermediate purification or desalted through PD-10 Desalting column (Cytiva Life Sciences, Marlborough, MA, USA; formerly GE Healthcare Life Sciences). At higher scale, the purification was done by Tangential Flow Filtration (TFF). The TFF was performed with a Sartorius Hydrosart 10 kDa cut-off membrane with a 200 cm^2^ membrane area. Fifteen volumes of diafiltration against water were performed (P_in_ 1.0 bar, P_out_ 0.0 bar, TMP 0.5 bar and permeate flow: 8–10 mL/min), keeping the retentate volume constant at 50 mL. The purified material, designated as GACox, was frozen at −80 °C and lyophilized. 

#### 4.3.2. Conjugation

GACox was conjugated to different carrier proteins (CRM_197_, SLO, SpyAD, SpyCEP) in borate buffer at pH 8 in the presence of NaBH_3_CN, with a GAC to protein to NaBH_3_CN ratio of 4:1:2 *w*/*w*/*w* and GACox concentration of 40 mg/mL. The reaction mixtures were incubated at 25 °C (for GAS proteins) or at 37 °C (for CRM_197_) for 2 days. Conjugates of GAS proteins were purified by Amicon Ultra (Merck, Darmstadt, Germany) 30 kDa cut-off against 10 mM NaPi pH 7.2 (3500× *g*; 4 °C; 8 washes). CRM_197_ conjugate was purified through anionic exchange chromatography on a 1 mL Sepharose Q FF column (Cytiva Life Sciences, Marlborough, MA, USA; formerly GE Healthcare Life Sciences): 1 mg of protein was loaded per mL of resin in 10 mM NaPi pH 7.2 and purified conjugate was eluted with a gradient of 1 M NaCl. Collected fractions were dialyzed against 10 mM NaPi pH 7.2 buffer. Final purified conjugates were designated as GACox-proteins.

After DoE optimization, the conditions were changed as following: GACox 40 mg/mL with GACox to CRM_197_ 1:1 *w*/*w* ratio, NaBH_3_CN 5 mg/mL, borate pH 8, ON at 25 °C. The reaction mixture was then diluted 10 times with PBS and NaBH_4_ (NaBH_4_:GAC *w*/*w* ratio of 0.5 to 1) was added to quench residual unreacted aldehydic groups of GACox [62]. The mixture was kept at RT for 2 h. Based on the scale, purification was done against PBS by Amicon Ultra 30 kDa cut-off as previously described or by TFF. The TFF was performed with a Sartorius PESU 50 kDa cut-off membrane with a 200 cm^2^ membrane area. Ten volumes of diafiltration against PBS 1M NaCl followed by 20 volumes of diafiltration against PBS alone were performed (P_in_ 0.5 bar, P_out_ 0.0 bar, TMP 0.25 bar, permeate flow rate: 25–27 mL/min), keeping the retentate volume constant at 50 mL. 

### 4.4. Design of Experiment (DoE)

Experimental planning and data elaboration were performed with Design-Expert 10, Stat-Ease Inc. Anderson-Darling normality test was performed using Minitab 18, Minitab Inc (State College, PA, USA).

For the oxidation step, each reaction test was performed on a total volume of 200 µL, purification was done through Vivaspin 3 kDa cut-off (Sartorius) against water. Oxidized GAC samples were assessed for % GAC recovery (based on Rha quantification by HPAEC-PAD), % GlcNAc oxidation and for GAC average chain length by HPLC-SEC analysis.

For the conjugation reaction, GAC was oxidized at 10 mg/mL with 8 mM NaIO_4_ in borate pH 8, for 30 min at 25 °C in the dark. After quenching of NaIO_4_ excess, the mixture was desalted by PD 10 against water and split in different vials for the conjugation runs. All conjugations were performed on a total volume between 20 and 50 µL and purified via Amicon Ultra 30 kDa cut-off against 10 mM NaPi at pH 7.2. Conjugates were assessed for % GAC recovery, GAC/CRM_197_ *w*/*w* ratios and % unconjugated CRM_197_ in the mixture.

For both DoE, the analyses were done following the same randomization scheme used to carry out the reactions.

### 4.5. Analytical Methods

Oxidized GAC was characterized by HPAEC-PAD [63] for evaluating % of GlcNAc oxidized, by comparing GlcNAc to rhamnose (Rha) molar ratios before (start) and after (ox) oxidation. The following equation was used, with all concentrations expressed as µmol/mL:

(1) $$\%\;\mathrm{GlcNAc}\;\mathrm{oxidation}\;=\;\left(1-\frac{\left[{\mathrm{GlcNAc}}_{\mathrm{ox}}\right]}{\left(\frac{\left[{\mathrm{GlcNAc}}_{\mathrm{start}}\right]}{\left[{\mathrm{Rha}}_{\mathrm{start}}\right]}\right)\times \left[{\mathrm{Rha}}_{\mathrm{ox}}\right]}\right)\times 100$$

HPLC-SEC was used for checking no changes in GAC chain length after oxidation.

Purified conjugates were characterized by micro BCA (Thermo Scientific, Waltham, MA, USA) and HPAEC-PAD [63] for total protein and total GAC content respectively and to determine the PS to protein ratios in the final products. GAC concentration from HPAEC-PAD analysis was determined based on Rha quantification, as GlcNAc is impacted in the oxidation step. Free GAC was quantified by HPAEC-PAD after its separation from the conjugate by conjugate co-precipitation with DOC [64]. The reaction mixtures, as well as the purified conjugates, were analyzed by SDS-PAGE to compare protein patterns of the conjugates with corresponding unconjugated proteins and by HPLC-SEC to verify conjugate formation (shift of the conjugate at higher MW compared to both unconjugated protein and saccharide). Finally, DSC analysis was used for evaluating GAS proteins and corresponding conjugates thermostability.

#### 4.5.1. Sodium Dodecyl Sulfate-Polyacrylamide Gel Electrophoresis (SDS-PAGE)

Tris-acetate gels 7% (NuPAGE, from Invitrogen, Waltham, MA, USA) were used for running SDS-PAGE analysis. The samples (5–20 µL with a protein content of 2–10 µg) were mixed with 0.5 M DTT (1/5, *v*/*v*) and NuPAGE LDS sample buffer (1/5, *v*/*v*). The mixtures were heated at 100 °C for 5 min. The gel, containing loaded samples, was electrophoresed at 45 mA in NuPAGE Tris-Acetate SDS running buffer (20×, Invitrogen, Waltham, MA, USA) and stained with Coomassie Blue Staining (Thermo Fischer, Waltham, MA, USA).

#### 4.5.2. High Performance Liquid Chromatography–Size Exclusion Chromatography (HPLC–SEC)

Conjugate, free protein and free GACox samples were eluted on a TSK gel G3000 PW_XL_ (30 cm × 7.8 mm) column (particle size 7 µm) with TSK gel PW_XL_ guard column (4.0 cm × 6.0 mm; particle size 12 µm) (TosohBioscience, King of Prussia, PA, USA). The mobile phase was 0.1 M NaCl, 0.1 M NaH_2_PO_4_, 5% CH_3_CN, pH 7.2 at the flow rate of 0.5 mL/min (isocratic method for 35 min). Sample volume of injection was 80 µL. Void and bed volume calibration was performed with λ-DNA (λ-DNA Molecular Weight Marker III 0.12–21.2 Kbp, Roche, Risch-Rotkreuz, Switzerland) and sodium azide (NaN_3_, Merck, Darmstadt, Germany), respectively. GACox peaks were detected by refractive index (RI). Protein and conjugate peaks were also detected using tryptophan fluorescence (emission spectrum at 336 nm, with excitation wavelength at 280 nm). For the Kd determination the following equation was used:

(2) $$\mathrm{Kd}\;=\;\frac{\left(\mathrm{Te}-T0\right)}{\left(\mathrm{Tt}-T0\right)}$$

where: Te = elution time of the analyte, T0 = elution time of the bigger fragment of λ-DNA and Tt = elution time of NaN_3_.

#### 4.5.3. Differential Scanning Calorimetry (DSC)

For DSC analysis the samples were prepared at a protein concentration of ~2–3 μM in 10 mM NaPi at pH 7.2. The DSC temperature scan ranged from 10 °C to 110 °C, with a thermal ramping rate of 150 °C per hour and a 5 s filter period. Data were analyzed by subtraction of the reference data for a sample containing buffer only. All experiments were performed in triplicate and mean values of the melting temperature (Tm) were determined.

### 4.6. Immunogenicity Studies in Mice

Mouse studies were performed at the Toscana Life Sciences Animal Facility (Siena, Italy), in compliance with the relevant guidelines (Italian D.Lgs. n. 26/14 and European directive 2010/63/UE) and the institutional policies of GSK. The animal protocols were approved by the Animal Welfare Body of Toscana Life Sciences and by the Italian Ministry of Health (AEC project No. 201309, approval date 20 December 2013;and 399/2017-PR, approval date 11 May 2017).

Female, 5 weeks old CD1 mice (8 per group) were vaccinated intraperitoneally (i.p.) with 200 µL of formulated antigens at study day 0 and 28. Approximately 100 µL bleeds (50 µL serum) were collected at day −1 (pooled sera) and at day 27 (individual sera) with final bleed at day 42.

Conjugates were formulated with 2 mg/mL Alhydrogel (Al^3+^). By SDS-PAGE silver staining analysis it was verified that >90% of the conjugates was adsorbed on Alhydrogel.

### 4.7. Assessment of Anti-GAC and Anti-GAS Carrier Protein Immune Responses in Mice

Pre-immune sera and individual mouse sera collected four weeks after the first and two weeks after the second immunization were analyzed for anti-GAC, -SpyCEP, -SLO and -SpyAD total IgG by enzyme-linked immunosorbent assay (ELISA) as previously described [65], with slight modifications. Briefly, mouse sera were diluted 1:100, 1:4000 and 1:160,000 in PBS containing 0.05% Tween 20 and 0.1% BSA. ELISA units were expressed relative to mouse anti-antigen standard serum curves, with best 5 parameter fit determined by five-parameter logistic equation. One ELISA unit was defined as the reciprocal of the standard serum dilution that gives an absorbance value equal to 1 in the assay. Each mouse serum was run in triplicate. Data are presented as scatter plots of individual mouse ELISA units and geometric mean of each group.

GAC-HSA (at the concentration of 1 µg/mL in carbonate buffer pH 9.6), SpyCEP, SLO and SpyAD (at the concentration of 2 µg/mL in carbonate buffer pH 9.6) were used as coating antigens. 

### 4.8. Flow Cytometry (FACS)

GAS strain GAS51∆M1 was grown overnight at 37 °C, in the presence of 5% CO_2_ in Todd Hewitt broth + Yeast extract (THY). Bacteria were pelleted at 8000× *g* for 5 min and washed with PBS. Bacteria were then blocked with PBS containing 3% (*w*/*v*) BSA for 15 min and incubated with mouse sera diluted in PBS + 1% (*w*/*v*) BSA (1:500, 1:5000 and 1:10,000) for 1 h. After washes with PBS, samples were incubated with Alexa Fluor 647 goat anti-mouse IgG (1:500) (Molecular Probes, Eugene, OR, USA) for 30 min. Finally, bacteria were fixed with 4% (*w*/*v*) formaldehyde for 20 min and flow cytometry analysis was performed using FACS Canto II flow cytometer (BD Biosciences, San Jose, CA, USA).

### 4.9. Functional Assays

#### 4.9.1. IL-8 Cleavage Inhibition Assay

Pre-immune and post-second immunization individual sera were tested in an IL-8 cleavage enzyme-linked immunosorbent assay (ELISA) assay to evaluate their ability to block SpyCEP proteolytic activity. The assay was performed as previously described [14] with some modifications. Briefly, SpyCEP (5 ng/mL) was preincubated with mouse polyclonal anti-SpyCEP serum at four different dilutions (1:100, 1:300, 1:900, 1:2700) for 5 min at 4 °C in PBS 0.5 mg/ml BSA. Pre-incubation of SpyCEP with buffer only and with pre-immune serum were used as negative controls. Then, human IL-8 (Gibco, Waltham, MA, USA; 10 ng/mL) was added and the reaction was incubated at 37 °C (reaction without enzyme was used as control). After 2 h, each reaction mix was diluted 20-fold and incubated in 96-well plates coated with a blend of monoclonal antibodies directed against distinct epitopes of IL-8 (Life Technologies, Waltham, MA, USA). The amount of IL-8 in each sample and in the control reaction (without the enzyme) was determined according to the manufacturer’s protocols, using a standard curve of IL-8. Each serum dilution was tested twice and the mean value with error bar was reported in the graph. Results are expressed as amount (ng/mL) of uncleaved IL-8 at each serum concentration tested. 

#### 4.9.2. In Vitro Hemolysis Assay

Pre-immune and post-second immunization individual sera were tested in a hemolysis assay to evaluate their ability to block SLO hemolytic activity. The assay was performed as previously described [14] with some modifications. Briefly, a red blood cell suspension was prepared by washing rabbit red blood cells (Emozoo, Casole D’Elsa, Italy) four times in PBS and resuspended in PBS (20% rabbit red blood cell suspension in PBS). Eight serial 2-fold dilutions of either anti-SLO sera or negative control pre-immune serum diluted in PBS with 0.5% BSA were prepared in 96 well round bottom plates then preincubated with 900 units/mL of SLO toxin (Merck, Darmstadt, Germany, diluted in PBS with 15 mM dithiothreitol, Invitrogen, Waltham, MA, USA) at RT for 30 min (in a final volume of 150 µL). Following addition of rabbit blood cell suspension (50 µL), incubation was continued for 30 min at 37 °C. Plates were finally centrifuged for 5 min at 1000× *g* and the supernatant was carefully transferred to 96-well flat-bottomed plates. The absorbance of the released hemoglobin was read at 540 nm. Each serum dilution was tested twice and the mean value with error bar was reported in the graph. Results are expressed as amount of hemoglobin (OD540) released by rabbit red blood cells at each serum concentration tested.

### 4.10. Statistics

Mann-Whitney two-tailed test was used to compare the immune response elicited by two different antigens, Kruskal-Wallis test with Dunn’s post hoc analysis was used for comparison among more than two groups. Wilcoxon test matched-pairs signed rank two-tailed test was performed to compare the response induced by the same antigen at day 27 vs. day 42.

## Figures and Tables

**Table 1:** Main characteristics of purified GAC conjugates with CRM_197_ and GAS proteins.

Conjugate	GAC/Protein Molar Ratio	GAC/Protein *w*/*w* Ratio
GACox-CRM_197_	7.2	0.86
GACox-SLO	3.3	0.36
GACox-SpyAD	8.2	0.64
GACox-SpyCEP	6.0	0.24

**Table 2:** Optimized conditions for GACox-CRM conjugation and predicted responses from the model, confirmed by performing an additional conjugation test.

Optimized Conditions	GAC/CRM_197_ *w*/*w*		GAC Recovery %	
	Predicted (95% CI for Mean)	Actual	Predicted (95% CI for Mean)	Actual
[GACox] = [CRM_197_] = 40 mg/mL; [NaBH3CN] = 10 mg/mL; borate buffer pH 8; T = 25 °C; 2 days reaction time	0.46 (0.39–0.52)	0.39	38 (32–43)	39

**Table 3:** Investigating role of NaBH_3_CN concentration and reaction time on GACox conjugation to CRM_197_.

[NaBH_3_CN] in Reaction (mg/mL)	Reaction Time	GAC/CRM_197_ *w*/*w* Ratio in Purified Conjugate
10	4 h	0.34
	ON	0.41
	2 days	0.39
5	4 h	0.36
	ON	0.46
	2 days	0.48
1	4 h	0.42
	ON	0.45
	2 days	0.47

**Table 4:** Conjugates produced at different scale in optimized conditions confirming expected results in terms of GAC to CRM_197_ ratio and process yield.

GAC/CRM_197_ *w*/*w*		GAC Recovery %	
Small Scale	Large Scale	Small Scale	Large Scale
0.44	0.51	44	39

## Supplementary Materials

Supplementary materials can be found at https://www.mdpi.com/1422-0067/21/22/8558/s1.

## Abbreviations

GAS	Group A *Streptococcus*
GAC	Group A Carbohydrate
SLO	Streptolysin O
LMICs	Low- and Middle-income Countries
RHD	Rheumatic Hearth Disease
GlcNAc	*N*-acetylglucosamine
PS	Polysaccharide
SDS-PAGE	Sodium dodecyl sulfate-polyacrylamide gel electrophoresis
MW	Molecular Weight
HPLC-SEC	High Performance Liquid Chromatography–Size Exclusion Chromatography
i.p.	Intraperitoneally
DSC	Differential Scanning Calorimetry
FACS	Flow cytometry
DoE	Design of Experiment
RT	Room Temperature
Rha	Rhamnose
HPAEC-PAD	Anion Exchange Chromatography coupled with Pulsed Amperometric Detection
TFF	Tangential Flow Filtration
ELISA	Enzyme-linked immunosorbent assay
