# Supplementary material for: Rational Design of a Glycoconjugate Vaccine against Group A Streptococcus
Source: Int J Mol Sci. 2020 Nov 13;21(22):8558. doi: 10.3390/ijms21228558 (PMC7696035; doi:10.3390/ijms21228558)
Supplement: Supplementary file 1 [file ijms-21-08558-s001.pdf]

Supplementary Materials

Rational Design of a Glycoconjugate Vaccine against Group A *Streptococcus*

Roberta Di Benedetto [1], Francesca Mancini [1], Martina Carducci [1], Gianmarco Gasperini [1], Danilo Gomes Moriel [1], Allan Saul [1,†], Francesca Necchi [1], Rino Rappuoli [2] and Francesca Micoli [1,*]

[1] GSK Vaccines Institute for Global Health (GVGH), Via Fiorentina 1, 53100 Siena, Italy; roberta.x.di-benedetto@gsk.com (R.D.B.); francesca.x.mancini@gsk.com (F.M.); martina.x.carducci@gsk.com (M.C.); gianmarco.x.gasperini@gsk.com (G.G); danilo.x.moriel-gomes@gsk.com (D.G.M); allan.saul@honorary.burnet.edu.au (A.S.); francesca.x.necchi@gsk.com (F.N.)

[2] GSK, Via Fiorentina 1, 53100 Siena, Italy; rino.r.rappuoli@gsk.com (R.R.)

* Correspondence: francesca.x.micoli@gsk.com; Tel.: +39-0577-53-9087

† Now retired.

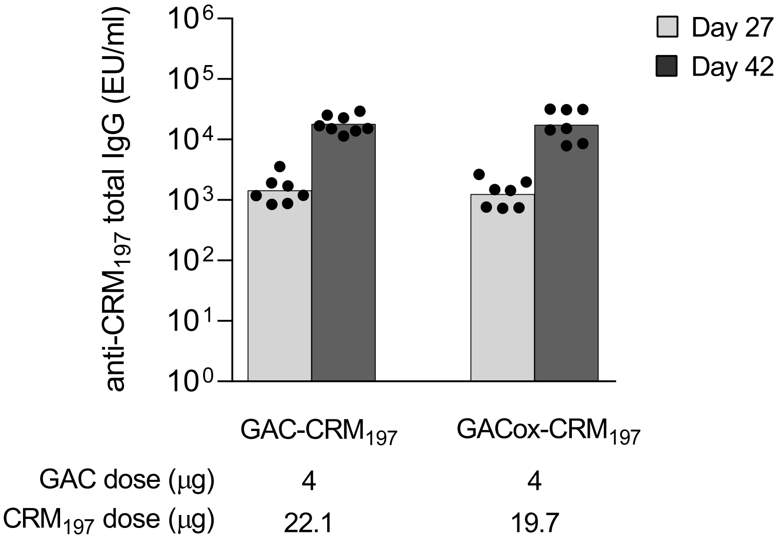

Figure S1. Immunogenicity of GAC when conjugated to CRM_{197} through different chemistries. CD1 mice were immunized i.p. at day 0 and 28 with 4 μg GAC/dose formulated with 2 mg/mL Alhydrogel. Summary graph of anti-CRM_{197} specific IgG geometric mean units (bars) and individual antibody levels (dots) is reported (CRM_{197} used as coating antigen). Mann-Whitney two-tailed test was performed to compare the response induced by the two immunization groups ($p > 0.05$).

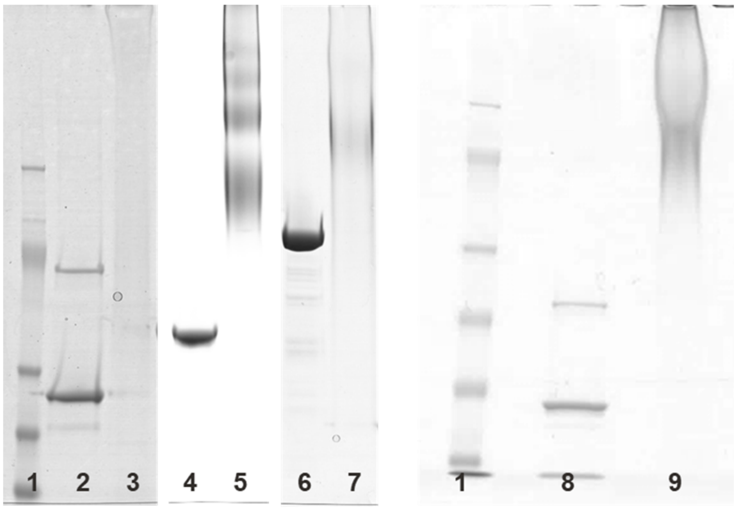

Figure S2. Characterization by SDS-PAGE analysis (3–8% Tris-acetate gel for GAS proteins conjugates, 7% Tris-acetate gel for CRM_{197} conjugate) of the conjugation mixtures in comparison to corresponding unconjugated proteins. Ten µg of conjugates and 2 µg of unconjugated proteins were loaded per well. Lane 1: marker, lane 2: SLO; lane 3: SLO conjugate; lane 4: SpyAD; lane 5: SpyAD conjugate; lane 6: SpyCEP; lane 7: SpyCEP conjugate; lane 8: CRM_{197}, lane 9: CRM_{197} conjugate.

Table S1. DoE approach applied to GAC oxidation: summary of conditions tested and results obtained.

Std	Run	Factor 1 A:[PS] mg/mL	Factor 2 B:[$NaIO_4$] mM	Factor 3 C:pH	Response 1 Recovery %	Response 2 Oxidation GlcNAc %	Response 3 Size Da
**5**	1	2.8	2.4	7.4	62	10.2	6970
**1**	2	2.8	2.4	5.6	69	13.5	6970
**20**	3	5.5	5.3	6.5	62	11.9	6782
**15**	4	5.5	5.3	6.5	76	12.7	6766
**2**	5	8.2	2.4	5.6	87	10.3	6886
**6**	6	8.2	2.4	7.4	78	8.5	6927
**16**	7	5.5	5.3	6.5	80	12.2	6807
**12**	8	5.5	10.0	6.5	88	16.7	6625
**4**	9	8.2	8.0	5.6	93	13.3	6825
**17**	10	5.5	5.3	6.5	78	12.8	6784
**14**	11	5.5	5.3	8.0	82	11.8	6808
**7**	12	2.8	8.0	7.4	77	14.4	6764
**3**	13	2.8	8.0	5.6	72	19.2	6766
**9**	14	1.0	5.3	6.5	70	19.4	6447
**11**	15	5.5	0.5	6.5	77	nd	7028
**8**	16	8.2	8.0	7.4	80	17.8	6726
**13**	17	5.5	5.3	5.0	84	17.8	6825
**18**	18	5.5	5.3	6.5	76	14.9	6774
**10**	19	10.0	5.3	6.5	88	15.1	6835
**19**	20	5.5	5.3	6.5	88	nd	6694

ANOVA for Response Surface Reduced Quadratic model

Analysis of variance table [Partial sum of squares - Type III]

Source	Sum of Squares	df	Mean Square	F Value	p-value Prob > F	
Model	134,14	5	26,83	9,33	0,0008	significant
A-[PS]	*15,35*	*1*	*15,35*	*5,34*	*0,0395*	
B-[NaIO4]	*73,85*	*1*	*73,85*	*25,67*	*0,0003*	
C-pH	*17,52*	*1*	*17,52*	*6,09*	*0,0296*	
AC	*14,61*	*1*	*14,61*	*5,08*	*0,0437*	
A^2	*19,97*	*1*	*19,97*	*6,94*	*0,0218*	
Residual	34,52	12	2,88			
Lack of Fit	*29,07*	*8*	*3,63*	*2,66*	*0,1798*	*not significant*
Pure Error	*5,46*	*4*	*1,36*			
Cor Total	168,67	17				

Std. Dev.	1,70	R-Squared	0,7953
Mean	14,04	Adj R-Squared	0,7100
C.V. %	12,08	Pred R-Squared	0,4656
PRESS	90,14	Adeq Precision	10,476
-2 Log Likelihood	62,80	BIC	80,15
		AICc	82,44

Factor	Coefficient Estimate	df	Standard Error	95% CI Low	95% CI High	VIF
Intercept	12,87	1	0,54	11,70	14,04	
A-[PS]	-1,06	1	0,46	-2,06	-0,060	1,00
B-[NaIO4]	2,65	1	0,52	1,51	3,78	1,01
C-pH	-1,13	1	0,46	-2,13	-0,13	1,00
AC	1,35	1	0,60	0,045	2,66	1,00
A^2	1,22	1	0,46	0,21	2,22	1,01

Figure S3. Identification of optimal conditions for GAC oxidation: statistical analysis of the model for the DoE.

Table S2. DoE approach applied to conjugation of GACox to CRM_{197}: summary of conditions tested and results obtained.

		**Factor 1**	**Factor 2**	**Factor 3**	**Response 2**	**Response 3**	**Response 4**
**Std**	**Run**	**A:[GACox]**	**B:[CRM_{197}]**	**C:[$NaBH_3CN$]**	**w/w Ratio GAC/CRM_{197}**	**Recovered PS**	**Unconjugated CRM_{197} in Mixture**
		**mg/mL**	**mg/mL**	**mg/mL**		**%**	**%**
**20**	1	25	25	25	0.26	21.1	0
**8**	2	40	40	40	0.28	20.7	0
**17**	3	25	25	25	0.29	23.1	0
**15**	4	25	25	25	0.29	22.6	0
**13**	5	25	25	10	0.42	35.4	0
**6**	6	40	10	40	0.49	9.4	0
**5**	7	10	10	40	0.16	12.8	0
**4**	8	40	40	10	0.43	41.3	0
**3**	9	10	40	10	0.12	41.2	33
**12**	10	25	40	25	0.34	41.9	0
**16**	11	25	25	25	0.30	22.2	0
**14**	12	25	25	40	0.25	18.9	0
**9**	13	10	25	25	0.15	32.9	0
**1**	14	10	10	10	0.27	22.0	5
**10**	15	40	25	25	0.49	25.6	3
**11**	16	25	10	25	0.47	12.5	0
**2**	17	40	10	10	0.65	9.2	16
**18**	18	25	25	25	0.33	26.1	0
**7**	19	10	40	40	0.12	30.8	11
**19**	20	25	25	25	0.34	27.0	0

(a) **Response** **w/w ratio GAC/CRM_{197}**

ANOVA for Response Surface Linear model

Analysis of variance table [Partial sum of squares - Type III]

**Source**	**Sum of Squares**	**df**	**Mean Square**	**F Value**	**p-value Prob > F**	
Model	0,32	3	0,11	43,45	< 0.0001	significant
A-[GAC]	*0,23*	*1*	*0,23*	*94,44*	*< 0.0001*	
B-[CRM_{197}]	*0,056*	*1*	*0,056*	*22,64*	*0,0002*	
C-[$NaBH_3CN$]	*0,033*	*1*	*0,033*	*13,28*	*0,0022*	
Residual	0,039	16	2,455E-003			
Lack of Fit	*0,035*	*11*	*3,153E-003*	*3,42*	*0,0926*	*not significant*
Pure Error	*4,608E-003*	*5*	*9,216E-004*			
Cor Total	0,36	19				

Std. Dev.	0,050	R-Squared	0,8907
Mean	0,32	Adj R-Squared	0,8702
C.V. %	15,32	Pred R-Squared	0,8084
PRESS	0,069	Adeq Precision	25,625
-2 Log Likelihood	-67,89	BIC	-55,91
		AICc	-57,23

**Factor**	**Coefficient Estimate**	**df**	**Standard Error**	**95% CI Low**	**95% CI High**	**VIF**
Intercept	0,32	1	0,011	0,30	0,35	
A-[GAC]	0,15	1	0,016	0,12	0,19	1,00
B-[CRM_{197}]	-0,075	1	0,016	-0,11	-0,041	1,00
C-[$NaBH_3CN$]	-0,057	1	0,016	-0,090	-0,024	1,00

Figure S4. *Cont.*

(b) **Response** **Recovered PS %**

ANOVA for Response Surface Linear model

Analysis of variance table [Partial sum of squares - Type III]

**Source**	**Sum of Squares**	**df**	**Mean Square**	**F Value**	**p-value Prob > F**	
Model	1640,06	3	546,69	31,69	< 0.0001	significant
A-[GAC]	*112,07*	*1*	*112,07*	*6,50*	*0,0215*	
B-[CRM_{197}]	*1209,63*	*1*	*1209,63*	*70,11*	*< 0.0001*	
C-[$NaBH_3CN$]	*318,36*	*1*	*318,36*	*18,45*	*0,0006*	
Residual	276,05	16	17,25			
Lack of Fit	*248,85*	*11*	*22,62*	*4,16*	*0,0638*	*not significant*
Pure Error	*27,20*	*5*	*5,44*			
Cor Total	1916,11	19				

Std. Dev.	4,15	R-Squared	0,8559
Mean	24,83	Adj R-Squared	0,8289
C.V. %	16,73	Pred R-Squared	0,7342
PRESS	509,31	Adeq Precision	21,521
-2 Log Likelihood	109,25	BIC	121,24
		AICc	119,92

**Factor**	**Coefficient Estimate**	**df**	**Standard Error**	**95% CI Low**	**95% CI High**	**VIF**
Intercept	24,83	1	0,93	22,86	26,80	
A-[GAC_{197}]	-3,35	1	1,31	-6,13	-0,56	1,00
B-[CRM]	11,00	1	1,31	8,21	13,78	1,00
C-[$NaBH_3CN$]	-5,64	1	1,31	-8,43	-2,86	1,00

Figure S4. Identification of optimal conditions for GACox conjugation to CRM_{197}: statistical analysis of the models for GAC/CRM_{197} w/w ratio (**a**) and GAC yield (**b**).
